# Intense Two-Octave Ultraviolet-Visible-Infrared Supercontinuum Laser via High-Efficiency One-Octave Second-Harmonic Generation

**DOI:** 10.34133/2022/9871729

**Published:** 2022-06-14

**Authors:** Mingzhou Li, Lihong Hong, Zhi-Yuan Li

**Affiliations:** School of Physics and Optoelectronics, South China University of Technology, Guangzhou 510641, China

## Abstract

Intense ultrabroadband laser source of high pulse energy has attracted more and more attention in physics, chemistry, biology, material science, and other disciplines. We report design and realization of a chirped periodically poled lithium niobate nonlinear crystal that supports ultrabroadband second-harmonic generation covering 350-850 nm by implementing simultaneously up to 12 orders of quasiphase matching against ultrabroadband pump laser covering 700-1700 nm with an average high conversion efficiency of about 25.8%. We obtain a flat supercontinuum spectrum with a 10 dB bandwidth covering more than one octave (about 375-1200 nm) and 20 dB bandwidth covering more than two octaves (about 350-1500 nm) in the ultraviolet-visible-infrared regime and having intense energy as 0.17 mJ per pulse through synergic action of second-order and third-order nonlinearity under pump of 0.48 mJ per pulse Ti:sapphire femtosecond laser. This scheme would provide a promising method for the construction of supercontinuum laser source with extremely broad bandwidth, large pulse energy, and high peak power for a variety of basic science and high technology applications.

## 1. Introduction

Ultrabroadband laser light sources have attracted more and more attention in physics, chemistry, biology, material science, and other disciplines [1–21]. However, the bandwidth that laser light sources can obtain is usually very limited. Optical supercontinuum generation (SCG) technology is a practical method to realize ultrabroadband spectral laser. At present, it has been widely used in various fields of modern scientific disciplines, such as optical frequency comb [6], precision frequency metrology [7], pulse compression [8], and optical coherence tomography [9]. In the past few decades, many important works on broadband SCG have been reported in systems including microstructure fiber [10–13], waveguide [14–16], and bulk [1, 2, 17, 18]. The most commonly used materials of SCG are silica, fluoride, chalcogenide glass, and other amorphous materials. Supercontinuum spectrum is generated by using the high peak power pump pulse of femtosecond or picosecond laser and the third-order nonlinear optical (3^rd^-NL) effects in materials, including four wave mixing, self-phase modulation (SPM), and stimulated Raman scattering [19–25]. In most reports, supercontinuum is mainly produced in the visible and infrared band. For the ultraviolet to visible band, one report shows that 110-400 nm UV-VUV laser can be generated by the combined action of nonlinear effect and dispersion in inflatable hollow fiber with the single pulse energy reaching 13 *μ*J, with the energy mainly concentrated in extreme ultraviolet [3]. Another report shows that by combining microstructure fiber and nonlinear crystal, supercontinuum output with nearly seven octave bands is obtained, with a spectral range of 340-40000 nm and a single pulse energy of 0.45 *μ*J [5].

However, the spectrum obtained only by third-order nonlinear effects has certain limitations. First, the spectral intensity far away from the central wavelength will be greatly reduced. Especially, for the supercontinuum spectrum covering a bandwidth with more than one octave, the intensity of the edge spectrum may be only -50 dB or even lower than the central wavelength. This leads to the very low flatness of supercontinuum. Second, SCG is often difficult to reach the short wave at ultraviolet band, which also limits its application. Third, for SCG based on fiber laser, the modal area is relatively small and the repetition rate of pump is large, so the pulse energy of SCG is hard to reach a high level.

To increase the pulse energy of SCG, high pulse energy femtosecond laser is injected into a long-path gas-filled hollow-core fiber [22–24] or multiple thin silica plate [25]. Besides, the two-color driving scheme [22, 24, 25], which injects both the fundamental and second-harmonic femtosecond pulse laser into the medium to ignite 3^rd^-NL effect, has been adopted both to increase the pulse energy and to expand the bandwidth. Although the pulse energy can reach the level of hundreds of *μ*J or even 1-2 mJ in these studies, the 20 dB bandwidth is still difficult to exceed two octaves [22–25]. Additionally, the spectral energy is mainly concentrated around the pump light, and the 10 dB bandwidth is usually difficult to exceed one octave. It can be said that although SCG covering a very broad bandwidth either in the ultraviolet-mid infrared (UV-MIR) range or in the ultraviolet-vacuum ultraviolet (UV-VUV) range has been realized successfully, it still remains a difficult and challenging task to accomplish supercontinuum laser encompassing ultraviolet-visible-infrared (UV-Vis-IR) spectral range with a two-octave bandwidth and simultaneously high pulse energy.

Another more popular scheme to expand the spectral range of laser is to utilize various second-order nonlinear optical (2^nd^-NL) effects, including second-harmonic generation (SHG), sum-frequency generation (SFG), difference-frequency generation (DFG), optical parametric oscillation, and amplification processes [20, 21, 26–38]. The quasiphase matching (QPM) scheme has been widely and deeply explored as a promising route [28–38]. By making the nonlinear coefficient of nonlinear crystal periodic, quasiperiodic, aperiodic, or chirped-periodic, additional reciprocal lattice vectors can be introduced into the nonlinear frequency conversion processes to compensate phase mismatch and accomplish high-performance laser frequency conversion and expansion. Significantly, chirped periodically poled lithium niobate (CPPLN) nonlinear crystals exhibit a series of discrete reciprocal lattice vector (RLV) bands with large effective nonlinear coefficients [34–38]. This series of discrete RLV bands can be used to meet not only broadband QPM SHG but also for simultaneous cascaded broadband SHG and SFG to create third-harmonic generation (THG) [36] and even high harmonic generation (HHG) [37], leading to the fascinating ultrabroadband Vis-NIR supercontinuum white laser but unfortunately with a low energy of only 2 *μ*J per pulse. This ultrabroadband QPM scheme also allows for collective operation and synergic action of 2^nd^-NL and 3^rd^-NL effects to create Vis-NIR supercontinuum white laser with a much higher energy of 30 *μ*J per pulse, broader bandwidth, flatter spectral profile, and interestingly, broadly tunable chroma [38]. Yet, the utilization of cascaded SHG and SFG to realize higher-order harmonics located in the short wavelength regime in previous works [36–38] might suffer from degraded energy conversion efficiency for higher-order HG compared with only the lowest order SHG [37, 38]. Thus, it would be interesting to find a scheme where only SHG works to create ultrabroadband supercontinuum lasers with bandwidth comparable with the scheme of cascaded SHG, THG, and HHG but with higher conversion efficiency.

In this work, we design and implement a CPPLN nonlinear crystal that exhibits multiple-order broadband RLV bands of QPM for direct SHG against a pump ultrabroadband NIR high-peak femtosecond laser. By controlling the duty cycle of poled period, up to 12 order QPM RLV bands are achieved simultaneously, covering a continuous RLV band. At each QPM order, the CPPLN crystal maintains a sufficiently large effective nonlinear coefficient and facilitates high-efficiency SHG against a segment of pump light spectral components. As a result, the designed CPPLN crystal enables high-efficiency direct SHG (instead of cascaded SHG and SFG) against the pump NIR laser with an extremely large bandwidth covering 700-1700 nm that comes from passing a high-peak power Ti:sapphire femtosecond laser through a fused silica. Then, we obtain a flat supercontinuum spectrum covering more than two octaves in a bandwidth of 20 dB, covering the UV-Vis-NIR range from 350 nm to 1500 nm, and reaching a high energy of 170 *μ*J per pulse. Moreover, the 10 dB bandwidth covers a wavelength range of 375-1200 nm, encompassing more than 800 nm and going well beyond one octave, which clearly represents a quite flat spectrum of supercontinuum laser.

## 2. Results

### 2.1. Design of CPPLN Nonlinear Crystal

**Figure 1:**
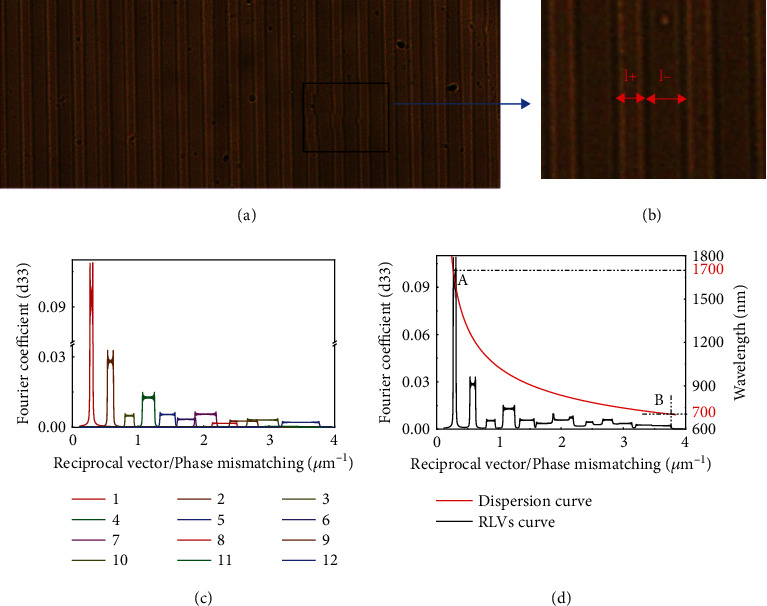
(a) Microscopic image of the fabricated sample surface of CPPLN structure. (b) The positive and negative domains of CPPLN with *D* = *l*_+_/(*l*_+_ + *l*_−_) = 0.36. (c) Calculated distributions of the nonlinear coefficient as functions of RLVs for duty cycle *D* = 0.36. Twelve RLV bands are shown, which correspond to 1st-12th order QPM. The nonlinear coefficients are expressed in units of the second-order nonlinear coefficient d_33_ of CPPLN, which is equal to 27.2 pm/V. (d) Combined plots of the phase-mismatch curves for SHG in a LiNbO_3_ crystal (right) and the nonlinear-coefficient curve for the CPPLN structure with the duty cycle of 0.36 (left). Points A and B denote the wavelength band (approximately from 700 nm to 1700 nm) in which SHG can occur simultaneously because of the fulfillment of the QPM condition, leading to simultaneous broadband SHG from this CPPLN structure.

Based on the principle of CPPLN design based on an effective nonlinear coefficient model [34, 35], we designed a CPPLN crystal which was suitable for ultrabroadband SHG, with the starting poled period *Λ*_0_ = 23.6 *µ*m, chirp date *D*_g_ = 2.4 *µ*m^−2^, and *z* = 2 cm, and the poled period was reduced from 23.6 *μ*m to 20 *μ*m. In addition, the LN crystal used was 5% MgO doped. The CPPLN sample was prepared via the standard electric poling technique on a z-cut LN with 2 mm thickness [36–38]. Figures 1(a) and 1(b) show the microscopic image of the fabricated sample surface, where the positive and negative domains are clearly visible.

For different values of duty cycle D, the effective nonlinear coefficients of each order QPM will be different. Figures 1(c) and 1(d) show the calculated distribution of G_*m*_(*k*) for a CPPLN nonlinear crystal with a duty cycle of *D* = 0.36, which are obtained by performing Fourier transform or Fourier series expansion on the spatial distribution of the second-order nonlinear coefficients of the designed CPPLN crystal. As shown in Figure 1(c), altogether 12 RLV bands can be recognized. The lowest 4 orders of RLV bands, separated by very narrow gaps, have a relatively high effective nonlinear coefficient, while the higher 5-12 RLV bands, connected with each other with no gap or even overlapping each other, have a relatively small effective nonlinear coefficient. Figure 1(d) displays the combined plot of all 1-12 RLV bands as well as the phase-mismatch curve for direct SHG in a wavelength range of fundamental-wave (FW) pump laser from 600 nm to 1800 nm. One can see that the CPPLN nonlinear crystal can provide a largely continuous RLV in a large frequency range, especially for higher wave vector mismatch corresponding to long wavelength. The lower order QPM RLV bands contribute to SHG for long-wavelength pump laser (see point A), while the higher order QPM RLV bands contribute to SHG for short-wavelength pump laser (see point B). The phase mismatch plot clearly shows that the designed and fabricated CPPLN can offer direct QPM SHG against the FW pump laser wavelength extending from 1700 nm to 700 nm and create SH signal laser covering a very broad bandwidth ranging from 350 nm in the UV band to 850 nm in the NIR band, with sufficiently high efficiency.

### 2.2. Experiment of SHG against One-Octave Pump Laser

**Figure 2:**
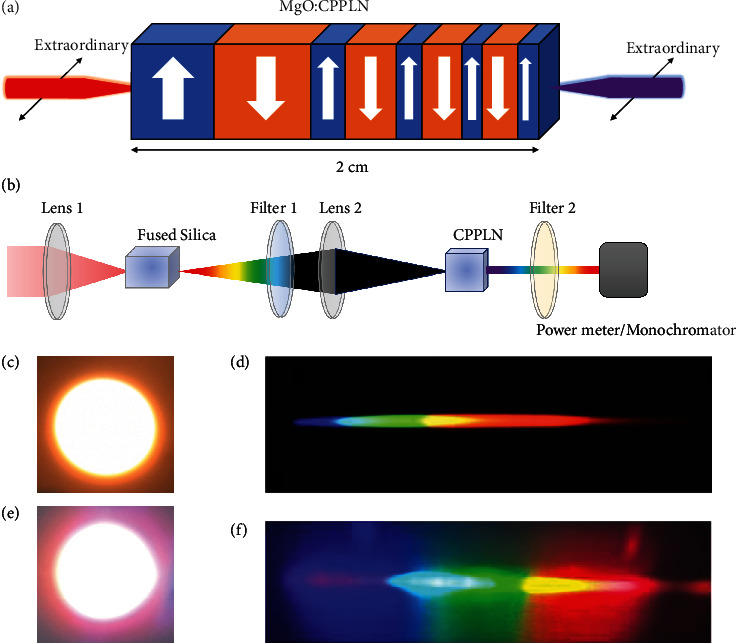
Schematic of the experimental setup. (a) The CPPLN sample illuminated by extraordinary FW pump laser to create SHG. Here, the poled period is greatly exaggerated for illustrative purposes. (b) Experimental setup of ultrabroadband SHG from the CPPLN sample. (c) The white light beam from the fused silica sample. (d) The 1st-order diffraction beam of (c). (e) The white light beam from the CPPLN sample. (f) The 1st-order diffraction beam of (e).

The principle of ultrabroadband SHG of CPPLN against an also ultrabroadband FW pump laser is schematically illustrated in Figure 2(a). According to the law of nonlinear optical frequency conversion, to accomplish the fascinating task and great mission of simultaneous direct SHG with ultrabroad bandwidth, two conditions must be satisfied. First, the input FW pump laser must have simultaneous ultrabroad bandwidth. Second, nonlinear crystal must also have ultrabroad operation bandwidth of SHG (i.e., with sufficiently high efficiency) comparable with the pump laser. Fairly speaking, these two conditions are not easy to satisfy. In fact, more precisely, it is very difficult to meet these two conditions simultaneously.

To see whether our designed CPPLN meets the harsh conditions and enables ultrabroadband SHG with fairly high efficiency, we designed an experimental setup illustrated in Figure 2(b) and used it to investigate ultrabroadband SHG against ultrabroadband FW pump laser and measure the quantitative values of conversion efficiency. To meet the first condition, we used the SCG scheme where a 2 cm thick fused silica was pumped by the Ti:sapphire femtosecond laser. This scheme is largely based on the well-established third-order nonlinearity (3rd-NL) self-phase modulation effect that accompanies automatically with the transport of high-peak power femtosecond pulse within solid materials like the current fused silica. The Ti:sapphire femtosecond laser (Coherent, Astrella USP) had a central wavelength of 780 nm, a pulse width 50 fs, a repetition rate 1 kHz, an averaged power 0.5 W (pulse energy 0.5 mJ), and a peak power density at the level of 3 GW/cm^2^ without focusing. The spectral profile and energy power of the supercontinuum light were monitored before the CPPLN sample and after the femtosecond pulse laser passes the fused silica and a long-pass filter stopping light below 700 nm. Then, the supercontinuum light was focused on the front surface of the CPPLN crystal through lens 2 to ignite SHG. To evaluate the CPPLN performance of ultrabroadband SHG, different filters were placed after the CPPLN sample to allow second-harmonic wave (SHW) in different wavelength ranges to pass. Finally, a power meter and monochromator were used to record the pulse power and make quantitative analysis of the SHG spectrum. Subsequently, the overall conversion efficiency and spectral profile of SHG were calculated and analyzed.

As shown in Figure 2(c), the white light beam was obtained from fused silica. For the reason that the supercontinuum from fused silica covers less component of short wavelengths, the color temperature of the light beam was warm. As shown in Figure 2(e), we observed a bright white light beam with good beam profile when the CPPLN was pumped by the supercontinuum light with the wavelength ranging in 700~1700 nm, which exhibits an even better beam profile due to high quality SCG due to 3^rd^-NL interaction of the pumped Ti:sapphire femtosecond pulse laser with fused silica plate. Compared with Figure 2(c), its color temperature was obviously cold because of more component of short wavelength. Then, we used a grating (1200 lines/mm) for a qualitative assessment of the involved spectral components. Figures 2(d) and 2(f) show the picture of the 1st-order diffraction beam of Figures 2(c) and 2(e). In Figure 2(d), the brightness of the diffraction beam was low. In Figure 2(f), obviously, the 1st-order diffraction beam contains the colors of all visible light, and the different colors are balanced. This preliminarily shows that the SHW spectrum generated by CPPLN contained uniform visible light components.

To explain reasonably the above experimental observation and confirm the scheme illustrated in Figures 2(a) and 2(b) can indeed meet the two harsh conditions of ultrabroadband SHG, we made a detailed analysis on the spectral profile and energy power in every step of light path in Figure 2(b). The setup in Figure 2(b) can be categorized into two cascaded parts. The first part is composed of the Ti:sapphire femtosecond laser and the fused silica plate, while the second part is composed of the CPPLN sample. Obviously, the first part is designated to serve as an ultrabroadband laser source, while the second part is assigned to convert the incoming long-wavelength FW pump laser from the first part into short-wavelength ultrabroadband SHG laser.

**Figure 3:**
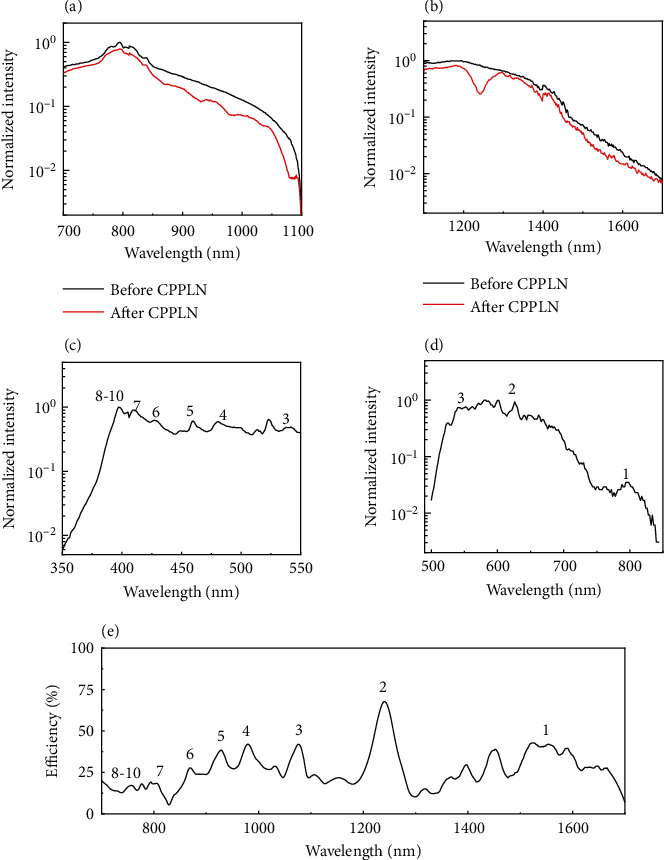
Spectrum of the supercontinuum measured before and after CPPLN sample at the wavelength of (a) 700-1100 nm and (b) 1100-1700 nm. Spectrum of the output SHW signals with the FW wavelength of (c) 700-1100 nm and (d) 1100-1700 nm. The peaks corresponding to various-order QPM are shown. (e) Measured conversion efficiencies for SHG as a function of the pumping wavelength for the CPPLN sample. 1^st^- to 10^th^-order QPM that corresponds to peaks of the conversion efficiency is shown.

The measured supercontinuum spectra before entering the CPPLN sample are shown by the black curves in Figures 3(a) and 3(b) for two segments, one between 700 and 1100 nm, the other between 1100 and 1700 nm, which were separately measured via a silicon detector and liquid nitrogen cooled InSb detector, respectively. Each spectrum has been normalized to its maximum spectral intensity. The total power of supercontinuum light in these two spectral segments was measured to be 87 and 10.2 mW, respectively. This result clearly shows that the first part of the optical setup depicted in Figure 2(b) can create an ultrabroadband laser source at least covering an extremely broad bandwidth between 700 and 1700 nm, indeed well meeting the first harsh condition towards our great mission.

We proceed to evaluate the performance of the CPPLN and see whether the second harsh condition of our great mission is satisfied. In order to avoid the overlapping between the spectra of FW and SHW and obtain accurate SHG efficiency, we used different filters to separate the supercontinuum laser into two parts for experiments, which are 700-1100 nm and 1100-1700 nm, respectively. The measured spectra of the SHG are presented in Figures 3(c) and 3(d) for two segments ranging within 350-550 nm and 550-850 nm, respectively. Here, the spectrum has been normalized against the maximum spectral intensity. The total power for these two spectral segments was measured to be 15.6 and 3.3 mW, respectively. A series of peaks appearing in the spectrum of SHG could be ascribed to the contribution from different QPM RLV bands, in good agreement with the QPM analysis illustrated in Figure 1(b). In the range of 700-1100 nm, the 1st-3rd order of QPM worked, and in the range of 1100-1700 nm, the 4th-10th order of QPM worked.

### 2.3. Efficiency of Ultrabroadband SHG

The ultrabroadband SHG via the CPPLN is also reflected from the energy depletion in the whole spectrum of FW pump laser. As clearly from Figures 3(a) and 3(b), the FW laser after passing through the CPPLN has its spectral intensity (red curves) reduced everywhere in the whole ranging covering 700-1700 nm with a considerable magnitude from the FW right before the CPPLN (black curves), indicating that SHG occurs efficiently for every spectral component in this extremely broad bandwidth. After careful evaluation and calibration considering the loss caused by Fresnel reflection of CPPLN, the conversion efficiency of SHG for different spectral components was obtained and presented in Figure 3(e), where the QPM order contribution is explicitly marked. The results are also in great agreement with the QPM analysis as shown in Figure 1(b). Here, a pronounced spectral continuity between the first and second order, different from the result of the theoretical calculation, may be due to the RLV band expansion of the domain structure during the fabrication of CPPLN sample. It can be seen that the SHG conversion efficiency of the CPPLN is mostly between 10% and 50% in the range of 350~850 nm, and the average efficiency calculated in this range is 25.8%.

We make a deeper analysis about the conversion efficiency for different QPM orders. It is a common knowledge from nonlinear optics that nonlinear conversion efficiency is related with the pump light spectral intensity and nonlinear coefficient. Although the 1st-order QPM has an effective nonlinear coefficient 3 times larger than that of the 2nd-order QPM (see Figure 1), the pump light spectral intensity for the 1st-order QPM (within wavelength 1400-1700 nm) is 10 times smaller than the 2nd-order QPM, and as a result, the SHG conversion efficiency corresponding to the 2nd-order QPM is higher than the 1st-order QPM. Similarly, as the pump light spectral intensity within 700-1100 nm and around the Ti:sapphire laser central wavelength 780 nm is much higher than the spectral intensity for wavelength above 1100 nm, the conversion efficiency is still remarkably high for these 4-10th order QPM SHG processes. Due to the compromised action between nonlinear coefficients and pump light spectral intensity, the CPPLN works with pretty high conversion efficiency over an extremely broad wavelength range of FW pumping light and enables the fascinating task of ultra-broadband direct SHG to be accomplished.

## 3. Discussion

**Figure 4:**
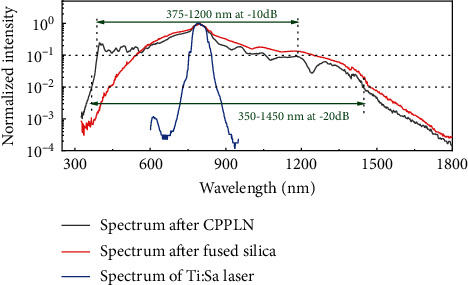
The spectra of supercontinuum after fused silica and CPPLN and the output spectrum of Ti:sapphire laser.

If we look upon the fused silica and CPPLN as a nonlinear optical module, then the cascaded 3rd-NL and 2nd-NL enabled by this module would convert the incoming pump Ti:sapphire femtosecond laser with a modest energy level of 0.5 mJ per pulse and a narrow spectral bandwidth of about 50 nm into a UV-Vis-NIR supercontinuum laser output encompassing more than two octaves' spectral bandwidth. As shown in Figure 4, based on the 3rd-NL self-phase modulation effect, the spectrum of Ti:sapphire femtosecond pulse is greatly broadened, but its intensity below the wavelength of 550 nm is obviously very weak. It is worth noting that after the CPPLN is added and 2nd-NL effect takes action, the 20 dB bandwidth of supercontinuum spectrum is increased significantly. The spectrum is extended to ultraviolet and becomes much flatter than before. Finally, the spectrum from ultraviolet to near infrared (375~1200 nm) is in the range of -10 dB, covering a bandwidth of about 800 nm, which is 16 times that of the pump Ti:sapphire femtosecond laser. In regard to the more popularly adopted -20 dB bandwidth standard, the bandwidth of the supercontinuum laser created by our silica-CPPLN module reaches more than two octaves, covering the UV-Vis-NIR range of about 350~1450 nm. The spectral bandwidth 1100 nm is about 22 times that of the pump Ti:sapphire femtosecond laser, which is a magnificently large number of bandwidth broadening. The result also clearly indicates that the synergic action of 2nd-NL and 3rd-NL effects would bring a superior power to build ultrabroadband supercontinuum laser far exceeding the single action of either 2nd-NL or 3rd-NL effects.

The current direct SHG scheme via CPPLN obviously has a much higher conversion efficiency than the previous scheme of cascaded SHG, SFG, and HHG via CPPLN in the creation of SCG, as reported in ref. [37]. The designed CPPLN has an unprecedented broad bandwidth of direct SHG, covering 700-1700 nm, together with a pretty high conversion efficiency. One reason from the success is that in the current scheme, we intentionally adopt a small effective nonlinear coefficient of CPPLN for the short-wavelength part (around 700-900 nm) and higher effective nonlinear coefficient for the long-wavelength part in order to reach a balanced output spectrum for SCG, because the incident pulse from silica plate has a much larger spectral intensity in the short-wavelength part than in the long-wavelength part. By using this balanced scheme in terms of pump pulse spectral intensity and effective nonlinear coefficient of CPPLN, we are able to obtain a flat spectral profile with a 10 dB bandwidth covering more than one octave (about 375-1200 nm) and 20 dB bandwidth covering more than two octaves (about 350-1500 nm) in the ultraviolet-visible-infrared regime and having intense energy as 0.17 mJ per pulse.

Due to the remarkably high conversion efficiency of CPPLN, the power level of the two-octave UV-Vis-NIR supercontinuum laser reaches a modest high level. The incoming Ti:sapphire femtosecond laser has a power level of 0.48 mJ per pulse, and after the fused silica plate, the supercontinuum laser has a power level of 0.255 mJ per pulse. Finally, after the CPPLN, the power of the output UV-Vis-NIR supercontinuum laser still maintains a high level as 0.17 mJ per pulse. Unfortunately, we have found that negative effect such as the nonlinear absorption and optical damage to materials will limit the further elevation of output laser power by simply increasing the power of pump femtosecond laser. These negative issues might be overcome by changing the infrastructures of the setup or by replacing silica with other materials with high damage threshold and lower nonlinear absorption.

In conclusion, we have successfully designed and implemented a CPPLN nonlinear crystal that supports ultrabroadband direct SHG covering 350-850 nm by adopting simultaneously up to 12 orders QPM against ultrabroadband pump laser covering 700-1700 nm, with an average high conversion efficiency of 25.8%. Magnificently, by sending a 0.48 mJ per pulse Ti:sapphire femtosecond laser through the nonlinear optical module composed of cascaded fused silica plate and CPPLN nonlinear crystal, we have accomplished a pretty flat supercontinuum spectrum with a 10 dB bandwidth covering the UV-Vis-NIR spectral range of 375-1200 nm and more than one octave bandwidth and with 20 dB bandwidth covering the UV-Vis-NIR spectral range of 350-1450 nm and more than two octaves' bandwidth, together with a modest high energy of 0.17 mJ per pulse. Our current scheme to create intense ultrabroadband laser sources of high pulse energy is simple, easy to carry out, stable, and free from complicated optical setups and infrastructures. It would provide a promising method for the construction of supercontinuum laser source with extremely broad bandwidth, large pulse energy, and high peak power. It can find potential applications in a variety of basic science and high technology areas such as large screen laser display, ultrabroadband tunable femtosecond optical parametric amplifier, supercontinuum laser source, optical combs, high-precision spectroscopy, and optical coherence tomography.

## 4. Materials and Methods

For the QPM technology, the nonlinear crystal has the nonlinear polarizability periodically, quasi periodically, or chirped periodically reversed along the propagation direction. After Fourier transform, it can correspond to the RVL distribution of different orders of QPM, and the effective Fourier coefficients corresponding to the RLVs of different orders are mainly related to the duty cycle D of positive or negative domains in poled period. For different orders of QPM, the crystal can meet the phase mismatch compensation for different wavelengths. For a periodically modulated nonlinear crystal like PPLN, the RLVs can be written as *K*_*m*_ = 2*π*/*Λ*_*m*_ (*m* is the QPM order, and *Λ*_*m*_ is the poled period equivalent to the *m*th-order QPM). When the fundamental wave at wavelength *λ*_1_ is converted to the second harmonic wave at wavelength *λ*_2_, the phase mismatch is Δ*k*_*q*_ = 2*k*_1_ − *k*_2_ − *K*_*m*_. If the phase mismatch is 0, the perfect quasiphase matching can be achieved. In comparison, for a chirped periodically modulated nonlinear crystal-like CPPLN, we can adopt a simplified local model where the crystal can be divided into many small segments and each segment can be approximately modeled by a PPLN with average period over the segment. Then, the equivalent poled period of different QPM orders can be approximately written as *Λ*_*m*_(*z*) = *Λ*(*z*)/*m* = 2*π*/*K*_*m*_ = (*l*_+_ + *l*_−_)/*m*. Here, *z* is the position of local region along the light propagation direction within the crystal, *Λ*(*z*) = *Λ*_0_/[1 + (*D*_*g*_*Λ*_0_*z*)/2*π*] is the local poled period of the crystal at position *z*. *Λ*_0_ is the starting poled period, *D*_*g*_ is the chirp rate, and *l*_+_ and *l*_−_are the local positive and negative domain width of the poled period. When the effective nonlinear coefficient of the medium is *d*_eff_, the local effective nonlinear coefficient along the *z* direction can be written in the form of Fourier series *d*(*z*) = ∑_*m*_*G*_*m*_exp(*iK*_*m*_*z*), where *G*_*m*_ is the Fourier coefficient. So, the effective nonlinear coefficient of each QPM order is *d*_m_(*z*) = *G*_*m*_exp(*iK*_*m*_*z*). In this way, one sees that *K*_*m*_ is also spatially varying. Then, the Fourier coefficient spectral curves of each order QPM order can be calculated and evaluated by performing Fourier transform or Fourier series expansion on the spatial distribution of nonlinear coefficient within the CPPLN crystal, which have been clearly demonstrated in Figures 1(c) and 1(d). In another more popular method, one performs Fourier transform over *d*(*z*) in the whole length of CPPLN and calculates Fourier coefficient as a function of the RLV. In this way, one can obtain the distribution of RLV bands corresponding to various QPM orders. Our numerical calculations show that the two methods yield the same results in regard to RLV distribution. Yet, the former method allows for easy classification of the QPM order (*m* = 1, 2, 3, ⋯) for each RLV band even when *m* > >1.

## Data Availability

The data that support the finding of this study are available from the corresponding author upon reasonable request.
